# Oncofetal markers CA 19-9, CA 125 and SP1 in healthy children and in children with malignancy.

**DOI:** 10.1038/bjc.1990.396

**Published:** 1990-11

**Authors:** M. Heikinheimo, J. Rajantie, P. Kuusela, M. J. Kallio, M. A. Siimes

**Affiliations:** Children's Hospital, Helsinki, Finland.


Br. J. Cancer (1990), 62, 865-867 © Macmillan Press Ltd., 1990

SHORT COMMUNICATION

### Oncofetal markers CA 19-9, CA 125 and SP1 in healthy children and in children with malignancy

M. Heikinheimo[1], J. Rajantie[1], P. Kuusela[2], M.J.T. Kallio[1] & M.A. Siimes[1]

[1]The Children's Hospital and [2]Department of Bacteriology and Immunology, University of Helsinki, Helsinki, Finland.

Urinary cathecholamines, serum neurone specific enolase, alpha-fetoprotein (AFP) and chorionic gonadotropin have a diagnostic role as tumour markers in children with malignancies. The new generation of tumour markers based on monoclonal antibodies has been extensively studied in adult patients but little is known about their occurence in children.

The CA 125 antigen is based on monoclonal antibodies originally raised against an ovarian serous cystadenocarcinoma cell line and it associates in serum with mucin fraction (Bast *et al.*, 1983). Serum CA 125 is elevated in about 80% of patients with ovarian cancer (Haglund, 1986), but it may also be elevated in patients with other gynaecological or gastrointestinal malignancies, and during pregnancy (Halila *et al.*, 1986).

CA 19-9 is a tumour marker raised against a human colorectal cell line (Koprowski *et al.*, 1979). CA 19-9 is immunohistochemically detectable in specimens from different gastrointestinal cancers or from normal pancreas, stomach, liver and gallbladder (Atkinson *et al.*, 1982; Haglund *et al.*, 1986*a*). The serum concentrations are elevated particularly in patients with pancreas cancer (Haglund *et al.*, 1986*b*; Herlyn *et al.*, 1982), but also in patients with other gastrointestinal cancer or some benign gastrointestinal diseases (Herlyn *et al.*, 1982).

Serum CA 19-9 and CA 125 measurements have not been shown to be of clinical value in extra-abdominal tumours, leukaemia or lymphoma of adult patients. This can be expected, since these markers cannot be histochemically located in the corresponding normal tissues.

Pregnancy-specific beta-1-glycoprotein (SP1) was originally isolated from human placenta (Bohn, 1971), but closely related antigens have subsequently been found in various human cell lines (Rosen *et al.*, 1979). SP1 is also shown to be a differentiation marker of the human myelomonocytic lineage (Heikinheimo *et al.*, 1987). These cells are capable of synthesising SP1. Serum concentration of SP1 has not been studied in disorders of the human haematopoietic system. SP1 is structurally homologous with carcinoembryonic antigen (Rooney *et al.*, 1988), another oncofetal antigen present in serum samples from cancer patients. Thus far, the clinical usefulness of SP1 has been associated with patients with trophoblast tumours (Rutanen *et al.*, 1980).

This study was undertaken to evaluate the role of serum concentrations of the two monoclonal tumour markers CA 125, and CA 19-9, and the oncoplacental protein SP1 in children with leukaemia and other malignancies.

Our study group consisted of 127 children with leukaemias or solid tumours, 51 girls and 76 boys, aged 0.2-16 years. Of the patients, 37 had acute lymphoblastic leukaemia, five had acute myeloid leukaemia, one had chronic myeloid leukaemia, 16 had lymphoma, ten had Wilm's tumour, 20 had neuroblastoma, 16 had brain tumour, nine had bone tumour and 13 had soft tissue sarcoma.

A serum sample was available from each patient at the time of diagnosis and from 63 patients at 1 to 8 weeks following diagnosis. In addition, serum samples from 52 healthy children, aged 1-17 years, collected for a nutritional study (Kallio *et al.*, 1989), were studied. Samples from 45 patients with acute infection without malignancy, and 12 children with active coeliac disease were available. The latter two groups were included since infections and various benign and malignant gastrointestinal diseases have been associated with elevated serum concentrations of the tumour markers (Herlyn *et al.*, 1982).

Serum concentrations of CA 125, CA 19-9, SP1 and AFP were measured using radioimmunoassays (Halila *et al.*, 1986; Haglund *et al.*, 1986*a*; Rutanen *et al.*, 1980; Ruoslahti & Seppälä, 1971). Statistical methods used were simple regression and correlation analyses and Student's *t* test.

The serum concentrations of CA 125, CA 19-9 and SP1 were similar in healthy individuals and in patients with acute infection or coeliac disease (Table I). The range of serum CA 19-9 levels in controls were clearly wider than in adults, and the values in some healthy individuals were more than $100\,U\,l^{-1}$. The analysis of the data showed that the age of individuals did not have any influence on the concentrations of the three markers. Further, the concentrations were also mostly within the range of the reference group in patients with leukaemia or solid tumours at diagnosis (Figure 1). However, a few individuals with malignancies had serum CA 125 and SP1 values marginally exceeding the upper limit of the reference values, as shown in Figure 1. The highest CA 125 levels were seen in three children with a large abdominal Burkitt's lymphoma causing partial intestinal obstruction in one of the patients. Involvement of the intestinal wall could, however, not be verified in these cases.

Clearly elevated serum CA 125 was also found in two children with acute T-cell leukaemia with intra-abdominal organ and nodal involvement as well as in one patient with stage II Wilm's tumour and in one patient with stage III retroperitoneal neuroblastoma. There was no relationship in general between any elevated marker levels and the extent of disease in different diagnostic groups.

In 15 of the 34 patients with elevated CA 125 ($n = 28$) and/or SP1 ($n = 8$) at diagnosis, follow-up samples were available at 1 to 6 weeks after diagnosis. In all these cases, the values decreased to below the upper limit of the reference values. On the other hand, follow-up samples were available for 48 cases with initial concentrations within the reference range. In ten of these patients the values were elevated above the reference range, although the change was not significant. All the patients, from which follow-up samples were available, received treatment for their disease. No difference in the response to the therapy was noted between patients whose marker level decreased, was stable or increased during the follow-up period.

Serum concentrations of CA 19-9, CA 125 or SP1 did not correlate to those of serum alanine aminotranspherase, aspartate aminotranspherase, creatinine, albumin, C-reactive protein or to the sedimentation rate. Neither did the marker levels correlate to each other or to the serum AFP values.

Correspondence: M. Heikinheimo.
Received 10 April 1990; and in revised form 4 June 1990.

**Table I** Serum CA 19-9, CA 125 and SP1 levels in healthy children and in patients with reactive conditions

| | Healthy children | | | Patients with | | | | | |
|---|---|---|---|---|---|---|---|---|---|
| | | | | Infections | | | Coeliac disease | | |
| | n | Median | Range | n | Median | Range | n | Median | Range |
| CA 19-9 (U $l^{-1}$) | 52 | 11 | <6.2–113 | 45 | 11 | <6.2–71 | 12 | 8.8 | <6.2–39 |
| CA 125 (U $l^{-1}$) | 52 | 12 | <5.6–25 | 41 | 8 | <5.6–68 | 12 | 14 | <5.6–39 |
| SP1 ($\mu$g $l^{-1}$) | 17 | <2.5 | <2.5–5.7 | 45 | <2.5 | <2.5–4.3 | 0 | | |

**Figure 1** Serum CA 19-9, CA 125 and SP1 concentrations in healthy children and in patients with malignancies. In the lymphoma group the symbol (O) illustrates patients with Burkitt's lymphoma. The light shaded area illustrates 90th percentile of control subjects values, and the dark shaded area the detection limit of the respective assay.

AFP levels were within the reference range in all 105 subjects studied.

This is the first detailed study of the serum levels of tumour markers CA 19-9, CA 125 and SP1 in healthy children and children with malignancies. The reference values for the paediatric age group were established.

Serum CA 19-9, CA 125 and SP1 concentrations are used in the detection of germ cell tumours in children and in the follow-up of these children (Heikinheimo *et al.*, 1986), but they are usually within normal limits in children with other malignancies. In some cases, however, the levels of CA 125 and SP1 are slightly elevated at diagnosis but normalise soon after the induction of therapy. Although SP1 has been shown to be a marker for the human myelocyte–monocyte lineage, children with acute myeloid leukaemia did not show elevated serum levels of SP1. The number of these patients was, however, low and further studies are needed to find out whether SP1 is released to the serum during the induction phase of the treatment in these patients.

In this study, the highest CA 125 values were noted in three patients with abdominal Burkitt's lymphoma. Unfortunately follow-up samples were not available from these patients. Our fourth patient with Burkitt's lymphoma, localised to the tonsils, had a normal serum CA 125, and elevation of this marker in three other patients may reflect the gastrointestinal involvement of the disease, and be unrelated to the type of the tumour. On the other hand, markedly elevated CA 125 levels were occasionally seen in other tumours, in which an abdominal involvement could not always be demonstrated. More patients with Burkitt's lymphoma should, however, be studied and followed up, to see if this tumour marker associates with tumour load and might thus have clinical importance.

The authors thank Dr Erkki Savilahti for providing the sera from coeliac patients, Ms Merja Helaterä and Ms Sirpa Kuisma for excellent technical assistance. This study was supported by the Paediatric Research Foundation and the Sigrid Juselius Foundation, Helsinki, Finland.

### References

ATKINSON, B.F., ERNST, C.S., HERLYN, M., STEPLEWSKI, Z., SEARS, H.F. & KOPROWSKI, H. (1982). Gastrointestinal cancer-associated antigen i immunoperoxidase assay. *Cancer. Res.*, **42**, 4821.

BAST, R.C. Jr, KLUG, T.L., ST JOHN, E. & 9 others (1983). A radioimmunoassay using a monoclonal antibody to monitor the course of epithelial ovarian cancer. *N. Engl. J. Med.*, **309**, 883.

BOHN, H. (1971). Detection and characterization of pregnancy proteins in the human placenta and their quantitative immunochemical determination in sera from pregnant women. *Arch. Gynek.*, **210**, 440.

HAGLUND, C. (1986). Tumour marker antigen CA 125 in pancreatic cancer: a comparison with CA 19-9 and CEA. *Int. J. Cancer*, **54**, 897.

HAGLUND, C., LINDGREN, J., ROBERTS, P.J. & NORDLING, S. (1986*a*). Gastrointestinal cancer-associated antigen CA 19-9 in histological specimens of pancreatic tumours and pancreatitis. *Br. J. Cancer*, **53**, 189.

HAGLUND, C., ROBERTS, P.J., KUUSELA, P., SCHEININ, T.M., MÄKELÄ, O. & JALANKO, H. (1986*b*). Evaluation of CA 19-9 as a serum tumour marker in pancreatic cancer. *Br. J. Cancer*, **53**, 197.

HALILA, H., STENMAN, U.-H. & SEPPÄLÄ, M. (1986). Ovarian cancer antigen CA 125 levels in pelvic inflammatory disease and pregnancy. *Cancer*, **57**, 1327.

HEIKINHEIMO, M., GAHMBERG, C.G., BOHN, H. & ANDERSSON, L.C.A. (1987). Oncoplacental protein $SP_1$ – a constitutive and inducible late differentation marker of the human myelomonocytic lineage. *Blood*, **70**, 1279.

HEIKINHEIMO, M., RAJANTIE, J., JALANKO, H., KUUSELA, P. & SIIMES, M.A. (1986). New tumor markers in childhood cancer. (Abstr.). *Pediatr. Res.*, **20**, 1045.

HERLYN, M., SEARS, H.F., STEPLEWSKI, Z. & KOPROWSKI, H. (1982). Monoclonal antibody detection of a circulating tumor-associated antigen. I. Presence of antigen in sera of patients with colorectal, gastric, and pancreatic carcinoma. *J. Clin. Immunol.*, **2**, 135.

KALLIO, M.J.T., SIIMES, M.A., PERHEENTUPA, J., SALMENPERÄ, L. & MIETTINEN, T.A. (1989). Cholesterol and its precursors in human milk during prolonged exclusive breast-feeding. *Am. J. Clin. Nutr.*, **50**, 782.

KOPROWSKI, H., STEPLEWSKI, Z., MITCHELL, K., HERLYN, D. & FUHRER, P. (1979). Colorectal carcinoma antigens detected by hybridoma antibodies. *Somat. Cell Genet.*, **5**, 957.

ROONEY, B.C., HORNE, C.H. & HARDMAN, N. (1988). Molecular cloning of a cDNA for human pregnancy-specific beta 1-glycoprotein: homology with human carcinoembryonic antigen and related proteins. *Gene*, **71**, 439.

ROSEN, S.W., KAMINSKA, J., CALVERT, I.S. & AARONSON, S. (1979). Human fibroblasts produce 'pregnancy-specific' beta-1-glycoprotein *in vitro*. *Am. J. Obstet. Gynecol.*, **134**, 734.

RUOSLAHTI, E. & SEPPÄLÄ, M. (1971). Development of radioimmunoassay for alpha-fetoprotein. Demonstration of alpha-fetoprotein in healthy human adults. *Int. J. Cancer*, **8**, 374.

RUTANEN, E.-M. & SEPPÄLÄ, M. (1980). Pregnancy-specific beta-1-glycoprotein in trophoblastic disease. *J. Clin. Endocrinol. Metab.*, **50**, 57.


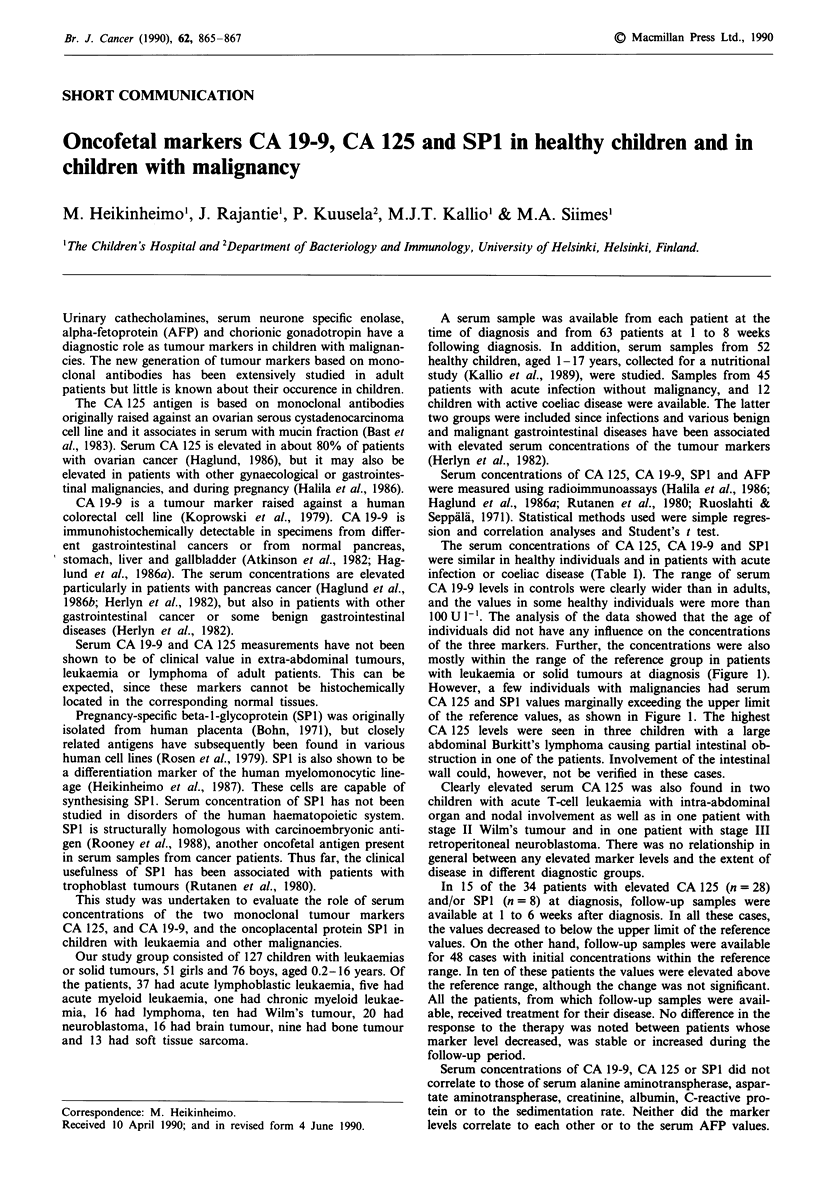

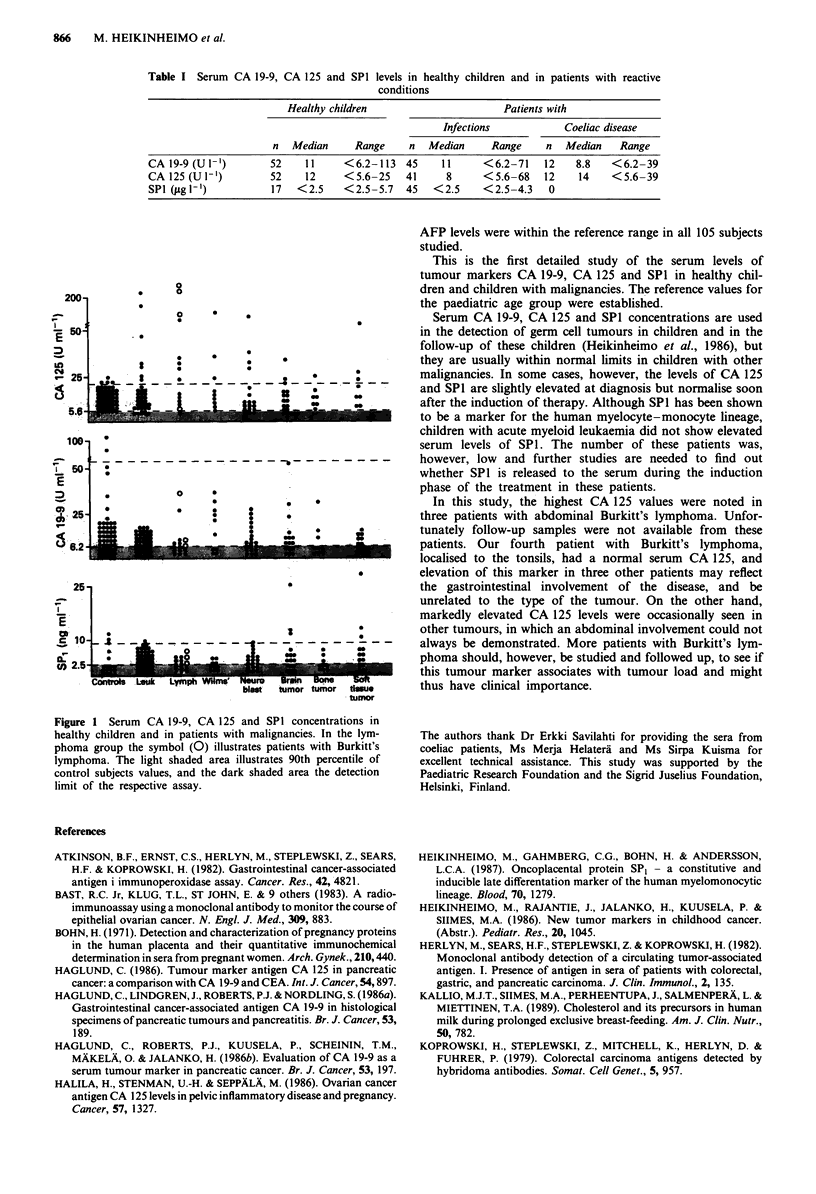

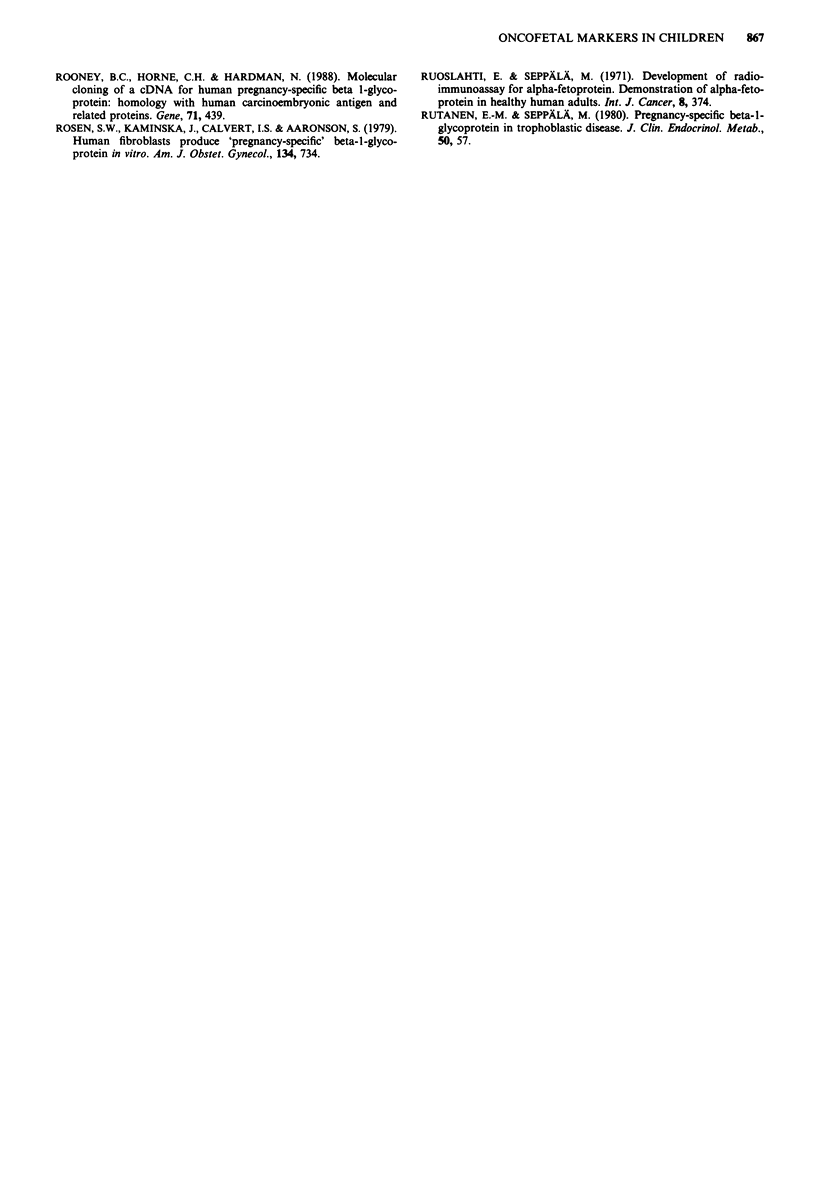


## References

[OCR_00246] Bast R. C., Klug T. L., St John E., Jenison E., Niloff J. M., Lazarus H., Berkowitz R. S., Leavitt T., Griffiths C. T., Parker L. (1983). A radioimmunoassay using a monoclonal antibody to monitor the course of epithelial ovarian cancer.. N Engl J Med.

[OCR_00258] Haglund C., Lindgren J., Roberts P. J., Nordling S. (1986). Gastrointestinal cancer-associated antigen CA 19-9 in histological specimens of pancreatic tumours and pancreatitis.. Br J Cancer.

[OCR_00264] Haglund C., Roberts P. J., Kuusela P., Scheinin T. M., Mäkelä O., Jalanko H. (1986). Evaluation of CA 19-9 as a serum tumour marker in pancreatic cancer.. Br J Cancer.

[OCR_00268] Halila H., Stenman U. H., Seppälä M. (1986). Ovarian cancer antigen CA 125 levels in pelvic inflammatory disease and pregnancy.. Cancer.

[OCR_00273] Heikinheimo M., Gahmberg C. G., Bohn H., Andersson L. C. (1987). Oncoplacental protein SP1--a constitutive and inducible late differentiation marker of the human myelomonocytic lineage.. Blood.

[OCR_00284] Herlyn M., Sears H. F., Steplewski Z., Koprowski H. (1982). Monoclonal antibody detection of a circulating tumor-associated antigen. I. Presence of antigen in sera of patients with colorectal, gastric, and pancreatic carcinoma.. J Clin Immunol.

[OCR_00290] Kallio M. J., Siimes M. A., Perheentupa J., Salmenperä L., Miettinen T. A. (1989). Cholesterol and its precursors in human milk during prolonged exclusive breast-feeding.. Am J Clin Nutr.

[OCR_00296] Koprowski H., Steplewski Z., Mitchell K., Herlyn M., Herlyn D., Fuhrer P. (1979). Colorectal carcinoma antigens detected by hybridoma antibodies.. Somatic Cell Genet.

[OCR_00303] Rooney B. C., Horne C. H., Hardman N. (1988). Molecular cloning of a cDNA for human pregnancy-specific beta 1-glycoprotein:homology with human carcinoembryonic antigen and related proteins.. Gene.

[OCR_00309] Rosen S. W., Kaminska J., Calvert I. S., Aaronson S. A. (1979). Human fibroblasts produce "pregnancy-specific" beta-1 glycoprotein in vitro.. Am J Obstet Gynecol.

[OCR_00314] Ruoslahti E., Seppälä M. (1971). Studies of carcino-fetal proteins. 3. Development of a radioimmunoassay for -fetoprotein. Demonstration of -fetoprotein in serum of healthy human adults.. Int J Cancer.

[OCR_00319] Rutanen E. M., Seppälä M. (1980). Pregnancy-specific beta-1-glycoprotein in trophoblastic disease.. J Clin Endocrinol Metab.

